# ASKθ, a group-III Arabidopsis GSK3, functions in the brassinosteroid signalling pathway

**DOI:** 10.1111/j.1365-313X.2010.04145.x

**Published:** 2010-02-25

**Authors:** Wilfried Rozhon, Juliane Mayerhofer, Elena Petutschnig, Shozo Fujioka, Claudia Jonak

**Affiliations:** 1 Gregor Mendel Institute of Molecular Plant Biology, Austrian Academy of SciencesDr. Bohr-Gasse 3, 1030 Vienna, Austria; 2 RIKEN Advanced Science Institute, Wako-shiSaitama 351–0198, Japan

**Keywords:** signal transduction, protein phosphorylation, GSK3/Shaggy-like kinase, brassinosteroid, bikinin, *Arabidopsis thaliana*

## Abstract

Brassinosteroids (BRs) are plant hormones that regulate many processes including cell elongation, leaf development, pollen tube growth and xylem differentiation. GSK3/shaggy-like kinases (GSK) are critical regulators of intracellular signalling initiated by the binding of BR to the BRI1 receptor complex. Three GSKs have already been shown to relay BR responses, including phosphorylation of the transcriptional regulator BES1. However, recent studies indicate that one or more yet unidentified protein kinases are involved in BR signalling. Here, we show that the *in vivo* protein kinase activity of the group-III GSK, ASKθ, was negatively regulated by BRI1. *Arabidopsis thaliana* plants with enhanced ASKθ activity displayed a *bri1*-like phenotype. ASKθ overexpressors accumulated high levels of brassinolide, castasterone and typhasterol, and were insensitive to BR. ASKθ localized to the nucleus and directly phosphorylated BES1 and BZR1. Moreover, the BES1/BZR1-like transcription factor BEH2 was isolated as an ASKθ interaction partner in a yeast two-hybrid screen. ASKθ phosphorylated BEH2 both *in vitro* and *in vivo*. Overall, these data provide strong evidence that ASKθ is a novel component of the BR signalling cascade, targeting the transcription factors BES1, BZR1 and BEH2.

## Introduction

Brassinosteroids (BRs) are polyhydroxylated plant steroid hormones. BRs regulate the expression of numerous genes, contribute to the regulation of cell elongation, division and differentiation, and help to control overall developmental programmes. They are also involved in regulating processes specific to plants, including pollen tube growth, vascular tissue development and photomorphogenesis (Clouse and Sasse, 1998). BR biosynthesis is well understood as a result of the identification and analysis of many BR-deficient mutants (Gachotte *et al.*, 1996; Kauschmann *et al.*, 1996; Li *et al.*, 1996; Szekeres *et al.*, 1996; Choe *et al.*, 1998, 1999a,b).

Plant steroids are perceived by transmembrane receptor kinases that initiate a phosphorylation-mediated signalling cascade. BRs bind to the extracellular leucine-rich repeats of the receptor kinase BRASSINOSTEROID-INSENSITIVE 1 (BRI1) and its close relatives BRI1-LIKE RECEPTOR KINASE 1 (BRL1) and BRL3 (Li and Chory, 1997; Cano-Delgado *et al.*, 2004; Zhou *et al.*, 2004; Kinoshita *et al.*, 2005). This leads to the phosphorylation of the cytoplasmic domain of BRI1 (Wang *et al.*, 2005a,b;), causing dissociation from the inhibitory protein BRI1 KINASE INHIBITOR 1 (BKI1) (Wang and Chory, 2006) and oligomerization with the co-receptor kinase BRI1-ASSOCIATED RECEPTOR KINASE 1 (BAK1), also known as SERK3 (Li *et al.*, 2002; Nam and Li, 2002; Russinova *et al.*, 2004; Wang *et al.*, 2005b; Yun *et al.*, 2009). Transphosphorylation of BRI1 and BAK1 was reported to enhance BR signalling (Clouse, 2008; Wang *et al.*, 2008; Yun *et al.*, 2009). The receptor can undergo endocytosis, which might be important for subsequent signalling (Russinova *et al.*, 2004; Geldner *et al.*, 2007).

Activation of the receptor kinases triggers a signalling pathway that is regulated by the GSK3/Shaggy-like protein kinase BRASSINOSTEROID INSENSITIVE 2 (BIN2; also known as ASKη, DWARF12 and UCU1) (Choe *et al.*, 2002; Li and Nam, 2002; Perez-Perez *et al.*, 2002). Like loss-of-function mutants in *BRI1*, *BIN2* gain-of-function mutants are BR-insensitive dwarfs with dark-green downward-curled leaves and with elevated levels of brassinolide, castasterone and typhasterol (Choe *et al.*, 2002; Li and Nam, 2002), indicating that BIN2 is a negative regulator of BR signalling. BIN2 phosphorylates and inhibits the BR-responsive transcription factors BRASSINAZOLE RESISTANT 1 (BZR1) and BRI1 EMS 1 (BES1; also known as BZR2) (He *et al.*, 2002; Yin *et al.*, 2002; Vert and Chory, 2006; Ryu *et al.*, 2007; Yan *et al.*, 2009).

BES1 and BZR1 belong to a plant-specific gene family of six closely related members, including BEH1–BEH4, which are dephosphorylated in response to BR (Yin *et al.*, 2005). All members of the BES1/BZR1 family possess eight adjacent repeats of the sequence SXXXS, which is a consensus GSK3 phosphorylation motif (Zhao *et al.*, 2002; Yin *et al.*, 2005). BIN2-mediated phosphorylation appears to regulate BZR1/BES1-like transcription factors by different mechanisms, including DNA-binding activity, protein stability and subcellular localization (He *et al.*, 2002; Yin *et al.*, 2002; Vert and Chory, 2006; Gampala *et al.*, 2007).

Recent data show that not only BIN2 but also ASKι and ASKζ are involved in BR signalling. Several lines of evidence indicate that one or more additonal GSK3/shaggy-like kinases convey BR signals (Vert and Chory, 2006; De Rybel *et al.*, 2009; Yan *et al.*, 2009). *Arabidopsis thaliana* possesses 10 GSK3/Shaggy-like kinases (ASKs), which can be grouped into four classes (Jonak and Hirt, 2002). BIN2, ASKι and ASKζ constitute group II. In an approach aimed at identifying novel functions of ASKs, we screened *ask* knock-out plants and seedlings with enhanced protein kinase activity of group-I, -III and -IV ASKs for various phenotypes. Interestingly, plants with enhanced ASKθ activity showed BR-related phenotypes resembling strong *bri1* alleles. Phenotypic and molecular analyses are presented, indicating a regulatory role for ASKθ in BR signalling. Biochemical data demonstrate that ASKθ activity is negatively regulated by BRI1, and that ASKθ phosphorylates the transcription factors BES1, BZR1 and BEH2 *in vitro* and in plant cells.

## Results

### Elevated ASKθ kinase activity induces severe dwarfism in *A. thaliana*

**Figure 1:**
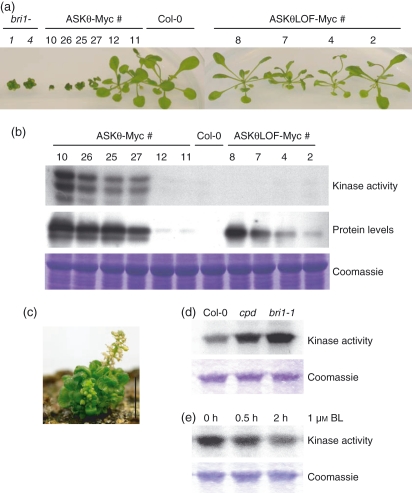
BRI1-regulated ASKθ activity modulates plant growth. (a) Phenotype of ASKθ overexpressor plants*. Arabidopsis thaliana* Col-0 plants expressing ASKθ-Myc or ASKθLOF-Myc under the control of the 35S promoter were grown for 21 days on half-strength MS plates containing 1% sucrose. Col-0, *bri1-1* and *bri1-4* were grown in parallel. (b) ASKθ-Myc kinase activity and protein levels of the transgenic lines shown in (a). ASKθ-Myc was immunoprecipitated with an anti-Myc antibody from 100 μg of protein extract, and ASKθ kinase activity was assayed with myelin basic protein as a substrate and [γ-^32^P]ATP as a co-substrate. Note that the batch of myelin basic protein used had three bands. ASKθ-Myc protein levels were detected by western blot analysis using an anti-Myc antibody from the same extract as used for the immunokinase assay. (c) Three-month-old plant of line ASKθ-Myc #25. The scale bar represents 1 cm. (d) Loss of BRI1 and CPD enhanced ASKθ kinase activity. Endogenous ASKθ was immunopreciptated from 100 μg of protein extract from 2-week-old plants. Subsequently, ASKθ kinase activity was determined by kinase assays with [γ-^32^P]ATP and myelin basic protein as a substrate. (e) Brassinolide inhibits ASKθ kinase activity. Two-week-old, *in vitro* grown Col-0 plants were transferred to liquid medium containing 1 μm brassinolide (BL) for the indicated period of time. ASKθ immunokinase activity was determined from 100 μg of protein extract. The autoradiogram in (e) was exposed for longer than in (d).

We generated *A. thaliana* transgenic lines expressing Myc-tagged versions of group-I, -III and -IV ASKs under the control of the strong 35S promoter. Seedlings of transformed lines showed a normal growth phenotype, except for plants transgenic for ASKθ-Myc. Plants overexpressing ASKθ-Myc showed extreme dwarfism, dark-green downward-curled leaves, low fertility and late flowering (Figure 1a,c). In addition, the hypocotyl was short and the cell length was remarkably diminished (Figure S1). Dark-grown plants showed partial de-etiolation (Figure S2). Thirty-nine of 101 independent ASKθ-Myc lines displayed this phenotype. The severity of the phenotype positively correlated with ASKθ-Myc protein levels and kinase activity. Plants exhibiting high ASKθ kinase activity were extremely dwarfed, whereas plants with moderate kinase activity showed a less severe phenotype (Figure 1a,b). Plants overexpressing a kinase-dead version of ASKθ, carrying the point mutation K167R in the ATP-binding pocket (ASKθLOF-Myc), looked like the wild type (Figure 1a). As expected, no ASKθ-Myc kinase activity was observed in these plants, although ASKθLOF-Myc protein was detected at similar levels as ASKθ-Myc (Figure 1b). These results indicate that ASKθ kinase activity is crucial for the development of the dwarf phenotype.

### ASKθ kinase activity is negatively regulated by BRI1

ASKθ overexpressor plants resembled *bri1* receptor mutants. To assess whether ASKθ might function downstream of BRI1, the *in vivo* kinase activity of ASKθ was analysed in wild-type and *bri1-1* plants. For this purpose, an ASKθ-specific peptide antibody (Figure S3) was generated and used for immunokinase assays. Wild-type plants showed low ASKθ activity, whereas ASKθ protein kinase activity was enhanced in *bri1-1* (Figure 1d). Similarly, endogenous ASKθ activity was increased in the BR biosynthesis mutant *cpd*, compared with wild-type plants (Figure 1d). Consistent with the genetic concept, ASKθ activity was downregulated by brassinolide (BR) (Figure 1e), showing that the protein kinase activity of ASKθ is negatively regulated by BR levels and by the BRI1 receptor complex.

### Plants overexpressing ASKθ are impaired in BR signalling

**Figure 2:**
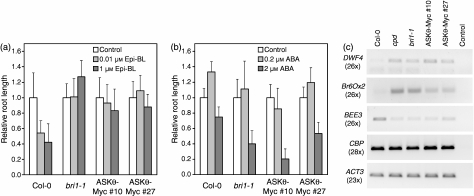
Plants overexpressing ASKθ are brassinolide insensitive. (a) Brassinolide insensitivity and (b) abscisic acid hypersensitivity. Seedlings were germinated and grown on plates for 7 days, and were subsequently transferred to plates containing the indicated concentrations of epi-brassinolide (Epi-BL) or abscisic acid (ABA). Root growth was measured 7 days after transfer. For each genotype, the root length was set to 1 for plants grown on control plates lacking hormones. The means and the standard deviations were calculated from at least 50 seedlings. (c) Semi-quantitative RT-PCR analysis of brassinosteroid (BR) marker genes. RNA was isolated from 2-week-old seedlings and analysed by semiquantitative RT-PCR. The numbers in brackets indicate the numbers of cycles used. *ACT3* (*At3g53750*) and *CBP* (*At5g44200*) were used as constitutively expressed controls. *DWF4* (*At3g50660*), *Br6Ox2* (*At3g30180*) and *BEE3* (*At1g73830*) are BR marker genes. A sample without template cDNA was used as a negative control.

To further characterize the effect of enhanced ASKθ activity, we selected lines #10 and #27, which have different ASKθ activity levels, and show either a strong or a mild phenotype, respectively, for further analyses. First, we analysed their response to epi-brassinolide and abscisic acid (ABA) in root growth assays (Figure 2a,b). Like *bri1* receptor mutants, plants overexpressing ASKθ-Myc were insensitive to epi-brassinolide but hypersensitive to ABA.

BR signalling regulates the expression of numerous genes. We analysed the transcript levels of marker genes for BR signalling and homeostasis, including the feedback-regulated BR biosynthesis genes *DWF4* and *Br6Ox2*, and the BR-transcription factor *BEE3* (Friedrichsen *et al.*, 2002; Coll-Garcia *et al.*, 2004; He *et al.*, 2005; Poppenberger *et al.*, 2005; De Rybel *et al.*, 2009). Like *bri1-1* and *cpd* plants, ASKθ-Myc overexpressor lines displayed increased *DWF4* and *Br6Ox2* expression, whereas *BEE3* transcript levels were reduced in these plants (Figure 2c).

To further assess the impact of ASKθ in the BR response pathway, we determined BR levels by GC-MS. Brassinolide, and its precursors castasterone and typhasterol, were dramatically elevated in lines overexpressing ASKθ-Myc compared with wild-type plants. Castasterone levels were 120-fold increased in ASKθ-Myc #10, were 21-fold increased in ASKθ-Myc #27 and were 75-fold increased in *bri1-1* mutants. Similarly, the levels of typhasterol were increased by a factor of 17, 6 and 4 in ASKθ #10, ASKθ #27 and *bri1-1*, respectively. ASKθ #10, ASKθ #27 and *bri1-1* contained 3.8, 0.5 and 1.2 ng of brassinolide per gram of fresh weight, respectively, whereas brassinolide was below the detection limit in Col-0 plants. The levels of these BRs positively correlated with the severity of the phenotype, abundance of the ASKθ-Myc protein and its kinase activity (Figure 1a,b). Taken together, these results indicate that BR signalling is disturbed in *A. thaliana* overexpressing ASKθ in a dose-dependent manner.

### ASKθ interacts with BEH2

**Figure 3:**
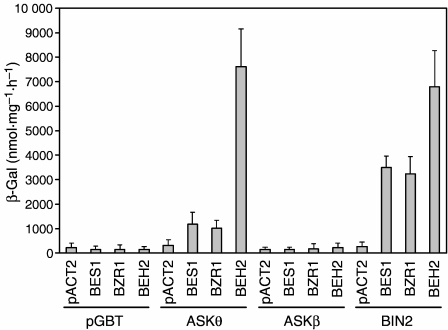
ASKθ interacts with BEH2. *Saccharomyces cerevisiae* strain PJ69-4A was co-transformed with the indicated combinations of ASKθ, ASKβ and BIN2 cloned into the binding domain vector pGBT9, and BES1, BZR1 and BEH2 were cloned into the activation domain vector pACT2. Empty vectors were used as controls. The β-galactosidase activity of protein extracts was measured using *o*-nitrophenyl-β-d-galactopyranoside (ONPG) as a substrate and calculated as nmol *o*-nitrophenol liberated in 1 h mg^−1^ of protein. The means and standard deviations were calculated from three independent experiments.

To identify substrates for ASKθ action, a yeast two-hybrid screen using ASKθ as bait was performed. Among the ASKθ-interacting clones, the full-length cDNA of the transcription factor BEH2 was isolated twice. BEH2 belongs to the BES1/BZR1 family of transcription factors involved in BR signalling (Yin *et al.*, 2005). The interaction of BEH2 with ASKθ was strong, as indicated by the growth of the yeast two-hybrid clones on medium without adenine. The specificity of interactions between ASKs and BES1/BZR1-like transcription factors was investigated with a quantitative β-galactosidase assay. ASKθ strongly interacted with BEH2 but also with BES1 and BZR1 (Figure 3). BIN2 interacted with BES1, BZR1 and BEH2. In contrast, ASKβ, the closest homologue of ASKθ, did not bind to any of the transcription factors tested.

### BEH2 and ASKθ localize to the nucleus

**Figure 4:**
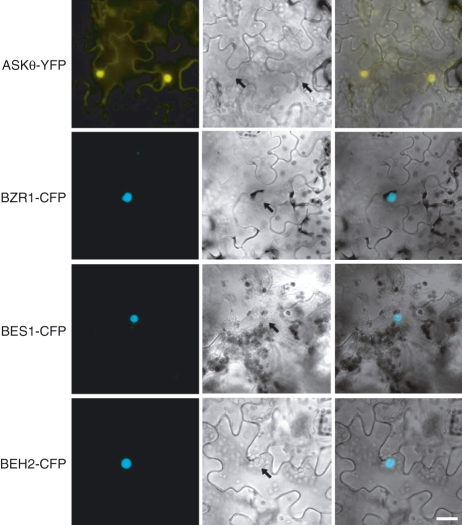
ASKθ and its putative substrates localize to the nucleus. YFP-tagged ASKθ and CFP-tagged versions of BZR1, BES1 and BEH2 were transiently expressed in *Nicotiana tabacum* leaves by infiltration with *Agrobacterium tumefaciens*. The subcellular localization of the proteins was observed by fluorescence microscopy (left). The nuclei are indicated by arrows in the bright-field pictures (middle). All pictures were taken at the same magnification. Scale bar: 20 μm.

In order to analyse whether ASKθ and BEH2 localize to the same subcellular compartment, their localizations were studied using protein fusions with YFP and CFP, respectively. CFP-tagged versions of BES1 and BZR1, which are known to be nuclear proteins (Yin *et al.*, 2002; Zhao *et al.*, 2002), served as controls. Constructs were transiently expressed in *Nicotiana tabacum* leaves by *Agrobacterium* infiltration. The fluorescence signals for the CFP-tagged transcription factors BES1, BZR1 and BEH2 were found in the nucleus. Although ASKθ does not have an obvious nuclear localization signal, ASKθ-YFP mainly localized to the nucleus, but low levels were also observed in the cytoplasm (Figure 4). Identical subcellular localization patterns were obtained when these proteins were transiently expressed in *A. thaliana* protoplasts or were stably expressed in *A. thaliana* plants (Figure S4 and data not shown).

### ASKθ directly phosphorylates BEH2, BZR1 and BES1

**Figure 5:**
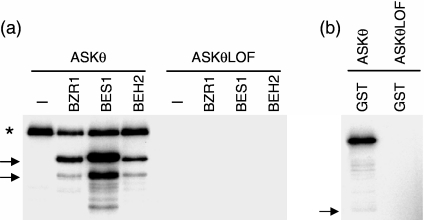
Direct phosphorylation of BZR1, BES1 and BEH2 by ASKθ. (a) Phosphorylation of BZR1, BES1 and BEH2 by ASKθ*in vitro*. Kinase assays were performed with purified GST-ASKθ or GST-ASKθLOF, and GST-BZR1, GST-BES1 or GST-BEH2. (b) ASKθ did not phosphorylate GST alone. The asterisk marks autophosphorylated ASKθ and the arrows indicate the position of the substrates.

The transcriptional activities of BZR1 and BES1 depend on their phosphorylation status (He *et al.*, 2002; Vert and Chory, 2006). To test whether ASKθ is able to phosphorylate BZR1, BES1 and BEH2, *in vitro* kinase assays were performed with recombinant proteins. GST-ASKθ phosphorylated GST-BZR1, GST-BES1 and GST-BEH2, but not GST alone (Figure 5). In addition, autophosphorylation of GST-ASKθ was observed, which was reduced in samples with the GST-tagged transcription factors, indicating that they were good substrates for ASKθ. In contrast, the kinase-dead version, ASKθLOF, showed neither activity towards the transcription factors nor autophosphorylation, confirming that active ASKθ is required for the phosphorylation of BZR1, BES1 and BEH2.

### BEH2, BZR1 and BES1 are phosphorylated by ASKθ*in vivo*

**Figure 6:**
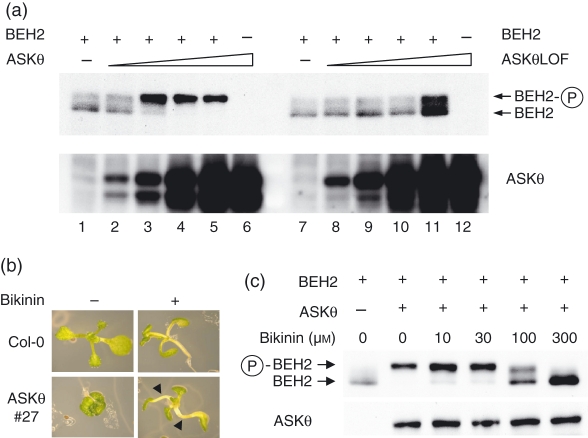
*In vivo* phosphorylation activity of ASKθ. (a) Phosphorylation of BEH2 by ASKθ. Arabidopsis protoplast cells were transformed with BEH2-CFP only or were co-transformed with increasing concentrations of either ASKθ-Myc or ASKθLOF-Myc. For BEH2-CFP, 5 μg of DNA was used; for ASKθ, 0.1 μg (lane 2), 0.3 μg (lane 3), 1 μg (lane 4) and 3 μg (lanes 5 and 6) were used; and for ASKθLOF, 0.1 μg (lane 8), 0.3 μg (lane 9), 1 μg (lane 10) and 3 μg (lanes 11 and 12) were transformed. BEH2-CFP was detected by western blot analysis with an anti-GFP antibody (upper panel). ASKθ-Myc was detected with an anti-Myc antibody (lower panel). The positions of the phosphorylated and unphosphorylated form of BEH2 are marked. (b) The dwarfed phenotype of an ASKθ overexpressor is rescued by bikinin. Plants were grown on half-strength MS plates (−) or on plates supplemented with 30 μM bikinin (+) for 14 days. Arrowheads indicate elongated petioles. (c) Phosphorylation of BEH2 by ASKθ inhibited by bikinin. Arabidopsis protoplast cells were transformed with BEH2-CFP or co-transformed with expression constructs for BEH2-CFP (5 μg per sample) and ASKθ-Myc (3 μg per sample). Proteins were detected by western blot analysis using an anti-GFP antibody for BEH2-CFP (upper panel) and an anti-Myc antibody for ASKθ-Myc (lower panel).

We then investigated whether ASKθ could phosphorylate BEH2 *in vivo*. *A. thaliana* protoplast cells were transformed with expression constructs of CFP-tagged BEH2 and increasing quantities of ASKθ-Myc. Proteins were detected by western blot analysis using tag-specific antibodies. A dominant, fast-migrating band and a slower, less intense band were observed in samples transformed with BEH2 alone (Figure 6a, upper panel, lanes 1 and 7), which is the typical pattern for BES1/BZR1-like transcription factors. The faster migrating band represents the unphosphorylated form, and the slower one represents the phosphorylated form (He *et al.*, 2002; Yin *et al.*, 2002, 2005; Vert and Chory, 2006). The upper band, seen in cells transformed only with BEH2, is likely to be the result of endogenous ASK activity. When ASKθ was co-expressed with BEH2, phosphorylated BEH2 accumulated in parallel with increasing quantities of ASKθ, whereas unphosphorylated BEH2 disappeared. Kinase-dead ASKθLOF had no effect on the ratio of phosphorylated and unphosphorylated BEH2, showing that ASKθ activity is necessary for BEH2 phosphorylation. Similar results were obtained when BZR1 and BES1 were used as substrates (Figure S5). These findings indicate that ASKθ phosphorylates BES1/BZR1-like transcription factors *in vitro* and *in vivo*.

### Bikinin rescues the ASKθ growth phenotype and inhibits ASKθ-mediated phosphorylation of BEH2

Bikinin, a small non-steroidal compound interfering with BR signalling by inhibiting the activity of ASK kinases, was recently identified in a chemical genetics screen (De Rybel *et al.*, 2009). Whereas brassinolide can only phenotypically rescue BR synthesis mutants, bikinin can also revert BR signalling mutants such as *bri1-116* and *bin2-1*. Similarly, bikinin partially rescued the growth phenotype of seedlings with enhanced ASKθ activity (Figure 6b). To assess the impact of ASKθ inhibition by bikinin on ASKθ target phosphorylation, protoplast cells were co-transformed with ASKθ-Myc and BEH2-CFP expression constructs. Aliquots of transformed cells were treated with increasing concentrations of bikinin, and the BEH2 phosphorylation status was determined by western blot analysis (Figure 6c). A predominant, fast-migrating band, representing unphosphorylated BEH2, was detected in cells transformed with BEH2 expression constructs. Co-expression of ASKθ induced the accumulation of the phosphorylated form of BEH2, which could be blocked in a dose-dependent manner by bikinin, providing further evidence that BEH2 is a target for ASKθ action.

## Discussion

Brassinosteroids (BRs) control a wide range of developmental and physiological processes. Extensive genetic screens have identified a number of components in BR synthesis and signalling that have led to substantial progress in elucidating the BR signalling cascade. However, because of a high level of redundancy, several important regulators appear not to be amenable to classical genetic approaches. In a dominant approach, we found that plants with elevated levels of ASKθ displayed a *bri1*-like phenotype. Hormone sensitivity tests, quantification of endogenous BR levels, expression analysis of marker genes and protein kinase assays indicate that ASKθ acts as a novel negative regulator in BR signalling.

Several lines of evidence show that the activity of ASKθ is a regulatory factor in BR signalling. The strict correlation of the BR-insensitive phenotypes with ASKθ kinase activity levels suggests that functional ASKθ kinase activity, rather than its protein level, is crucial for tuning BR signal transduction. Plants with constitutively high ASKθ activity had a severe phenotype, whereas plants showing moderate ASKθ kinase activity displayed a mild phenotype. In contrast, plants expressing large levels of a kinase-dead version of ASKθ did not exhibit any obvious phenotype. In line with the biochemical analyses, inhibition of ASKθ activity by bikinin reverted the phenotype of plants overexpressing kinase-active ASKθ.

Significantly, endogenous ASKθ activity was elevated in *cpd* plants, in which BR synthesis is defective, and ASKθ activity was downregulated by BR. Moreover, the activity of ASKθ was enhanced in *bri1-1* plants, providing strong evidence that ASKθ functions downstream of the BRI1 receptor complex, and is negatively regulated by BRI1-dependent BR-stimulated signalling. Analysis of the gain-of-function mutant *bin2-1* and of BR-dependent BIN2 activity supports the notion that the GSK kinase activity level is central for BR signal transduction (Li and Nam, 2002; Vert and Chory, 2006; Peng *et al.*, 2008).

In animal signal transduction pathways, GSK3 activity is regulated by protein phosphorylation and differential complex formation (Cohen and Frame, 2001; Woodgett, 2001; Jope and Johnson, 2004). Recent results indicate that plant GSK activity is highly controlled by different mechanisms. Wound-induced GSK (WIG) is activated by a post-translational mechanism, and WIG inactivation involves transcription and translation (Jonak *et al.*, 2000). ASKs are *in vivo* phosphoproteins and tyrosine phosphorylation in the T-loop is essential for its kinase activity (de la Fuente van Bentem *et al.*, 2008). Thus, it will be of great interest to elucidate whether the recently indentified class of BR-signalling kinases (BSKs) (Tang *et al.*, 2008) is able to phosphorylate and modulate BR-responsive GSKs.

Plant GSKs are also regulated by the proteasome (Wrzaczek *et al.*, 2007; Peng *et al.*, 2008), and the gain-of-function mutation of *bin2-1* appears to stabilize BIN2 (Peng *et al.*, 2008). In this respect, it is worth noting that overexpression of wild-type ASKθ (but not of the other nine ASKs; WR,EP,CJ, unpublished data) was sufficient to obtain the typical BR dwarfism. This might point towards a different regulation of ASKθ. Alternatively, the constant supply of ASKθ by the strong constitutive 35S promoter might abrogate the reduction of ASKθ activity and protein levels by BR.

Similar to BIN2, ASKθ might link BR signalling to transcription factors. ASKθ-YFP was predominately localized to the nucleus, which is in agreement with previous *in situ* immunostaining studies (Tavares *et al.*, 2002). Plants overexpressing ASKθ displayed a gene expression pattern similar to *bri1* receptor mutants (Figure 2c; JM, CJ, unpublished microarray data). Moreover, BES1, BZR1 and BEH2 appear to be cellular targets of ASKθ action. ASKθ interacted with BES1, BZR1 and BEH2. ASKθ was able to directly phosphorylate recombinant BES1, BZR1 and BEH2, and, importantly, ASKθ activity induced phosphorylation of these transcription factors in a dose-dependent manner in protoplast cells. Phosphorylation of BES1 prevents BES1 from binding DNA, and abolishes its transcriptional activity (Yin *et al.*, 2005; Vert and Chory, 2006). Future analyses will assess whether ASKθ modulates BES1, BZR1 and BEH2 activity by a similar mechanism.

Three GSK3/shaggy-like kinases, BIN2, ASKι and ASKζ, have previously been shown to be involved in mediating the BR response (Vert and Chory, 2006; Yan *et al.*, 2009). However, based on phenotypical and biochemical data, it was hypothesized that a yet unidentified GSK3 plays an important role in BR signalling. *bin2 askι askζ* knock-out mutants only display a mild phenotype, and a considerable fraction of BES1 is still phosphorylated in this triple mutant, indicating the involvement of one or more additional protein kinases in the phosphorylation of BES1/BZR1-like transcription factors. As ASKθ is able to phosphorylate BES1, it will be interesting to investigate whether BES1 is completely unphosphorylated in *bin2 askι askζ askθ* quadruple mutants, or whether yet additional protein kinases target BES1/BZR1-like transcription factors.

Overall, our data confirm and extend the notion that GSKs are central players in BR signalling. We identified the group-III GSK, ASKθ, as a novel regulator of BR signalling. Our data indicate a model in which ASKθ acts in a BR-regulated manner downstream of BRI1 to phosphorylate BES1/BZR1-like transcription factors. The normal growth phenotype of knock-outs of ASKθ (Claisse *et al.*, 2007) suggests that ASKθ works in parallel with BIN2, ASKι and ASKζ in the BR response pathway. There appears to be a high degree of redundancy at various levels in BR signalling. On the other hand, ASKθ, BIN2, ASKι and ASKζ have overlapping but distinct expression patterns (Dornelas *et al.*, 1999), and ASKθ and BIN2 displayed different binding specificities towards the distinct members of the family of BES1/BZR1-like transcription factors in yeast interaction assays (Figure S6), pointing towards specific functions of ASKθ, BIN2, ASKι and ASKζ. Thus, to better understand the fine tuning of the BR signalling network, it will be important to quantify the binding affinities between different GSKs and BES1/BZR1-like transcription factors *in planta*, and to unravel the role of the individual BR-regulated GSKs in different plant tissues throughout development.

## Experimental procedures

### Plant cultivation

*Arabidopsis thaliana* Col-0 plants were grown under long-day conditions (16-h light/8-h dark) with 80 μE m^−2^ s^−1^ cool white light. For *in vitro* cultures, plants were grown on half-strength MS medium (Duchefa, http://www.duchefa.com) supplemented with 1% sucrose.

### Generation of transgenic lines

The cDNAs of *ASKθ*, *BES1*, *BZR1* and *BEH2* were cloned into the binary plant expression vector, pGWR8, under the control of the 35S promoter. pGWR8, a derivative of pGreenII (Hellens *et al.*, 2000), carries the translational leader of the tobacco etch virus for efficient translation. cDNAs were tagged with the Myc epitope or YFP. The constructs were transformed into *Agrobacterium tumefaciens*GV3101/pSoup by electroporation (Hellens *et al.*, 2000). Transgenic *A. thaliana* Col-0 lines were generated by the floral-dip method (Clough and Bent, 1998), and were then selected on kanamycin-containing medium (50 μg ml^−1^).

### Western blot analysis

Equal quantities of proteins were separated by 10 or 15% SDS-PAGE and blotted onto polyvinylidene difluoride membranes (Millipore, http://www.millipore.com). The membranes were probed with anti-Myc (A-15; Santa-Cruz Biotechnology, http://www.scbt.com) or anti-GFP (Roche, http://www.roche.com) antibodies. Alkaline phosphatase-conjugated goat anti-rabbit IgG (Santa Cruz Biotechnology) or goat anti-mouse Fab-specific fragment (Sigma-Aldrich, http://www.sigmaaldrich.com) were used as secondary antibodies. The reaction was detected by chemiluminescence using the CDP-Star™ detection reagent (Amersham Biosciences, now part of GE Healthcare, http://www.gelifesciences.com).

### Kinase assays

Immunokinase and *in vitro* kinase assays were performed as described previously (Jonak *et al.*, 2000; De Rybel *et al.*, 2009).

### Physiological analysis

For hormone sensitivity assays, seeds were germinated on half-strength MS supplemented with 1% sucrose for 7 days. Subsequently, seedlings were transferred to plates supplemented with phytohormones of the indicated concentrations (Figure 2) or control plates. Plants were grown vertically and root growth was measured after 7 days. For each hormone concentration, 50–75 seedlings were measured and the root growth relative to control plates was calculated.

### BR level measurements

Brassinosteroid (BR) levels were quantified as described previously (Fujioka *et al.*, 2002).

### Yeast two-hybrid screen and quantitative interaction assay

A two-hybrid screen was performed with ASKθ as the DNA-binding bait, cloned in the vector pGBT9-Cam. The screen was carried out in yeast strain PJ69-4A using an *A. thaliana* root/leaf tissue cDNA library and an *A. thaliana* cell culture cDNA library (Farras *et al.*, 2001). Transformants (1.6 × 10^6^) were screened for histidine prototrophy on minimal medium containing 5 mm 3-amino-1,2,4-triazole. Library plasmids were obtained by transformation of yeast DNA into *Escherichia coli* XL1-blue and selection on LB-Amp (100 μg ml^−1^) agar plates. Interactions were confirmed by transformation of the rescued plasmids into *Saccharomyces cerevisiae* PJ69-4A containing the bait construct, and assaying for histidine and adenine prototrophy. Quantitative two-hybrid assays were performed as described previously (Teige *et al.*, 2004). The activity was calculated as nmol *o*-nitrophenol released per milligram of protein per hour. The protein concentration of the cell extracts was measured with the Bradford method using a kit from Carl Roth (http://www.carlroth.com).

### Transient expression in *Nicotiana tabacum* leaves

YFP- and CFP-tagged versions of ASKθ, BEH2, BES1 and BZR1 were transiently expressed in tobacco leaves by a previously described method (Yang *et al.*, 2000). Infiltrated leaves were examined by fluorescence microscopy using a Zeiss AX10 microscope (Zeiss, http://www.zeiss.com) equipped with a SPOT Pursuit camera (Diagnostic Instruments, http://www.diaginc.com).

### Transient expression in *A. thaliana* protoplasts

Transient expression assays were performed with protoplasts prepared from an *A. thaliana* cell suspension culture. Isolation, transformation and cultivation of protoplasts have been described previously (Cardinale *et al.*, 2002). CFP-tagged versions of BEH2, BES1 or BZR1 were used for co-transformation with different quantities of Myc-tagged ASKθ or ASKθLOF. In some experiments, MG115 was added at a concentration of 50 μM to stabilize the proteins. Bikinin was added in different concentrations to inhibit the ASKθ kinase activity. At 12–16 h after transformation, total protein extracts were prepared from protoplasts by the addition of SDS-PAGE loading buffer and boiling for 2 min. Western blot analysis was performed as described above.

### RT-PCR

RNA was isolated from 2-week-old seedlings using a Qiagen RNeasy kit (Qiagen, http://www.qiagen.com). A 2-μg portion of RNA was reverse-transcribed with M-MuLV reverse transcriptase (Fermentas, http://www.fermentas.com), as recommended by the manufacturer. A 2-μl portion of the reaction product was used as a template for semiquantitative PCR with Taq DNA polymerase (Fermentas) and gene-specific primers (Table S1). The amplification products were analysed by agarose gel electrophoresis.

## SUPPORTING INFORMATION

**Figure S1.** The cell size of plants overexpressing ASKθ is not affected by Epi-BL application. Plants were grown on ½ MS medium without (−BL) or supplemented with 1 μm epi-brassinolide (+BL) for 2 weeks and pictures of epidermal hypocotyl cells were taken. The cell size of *cpd* mutants was increased by application of epi-brassinolide, while *bri1-1* mutants and ASKθ-Myc overexpressor plants were not affected. All pictures were taken at the same magnification. The size bar represents 20 μm.

**Figure S2.** Phenotype of dark-grown ASKθ overexpressor plants. Col-0, *bri1-1*, *cpd* and ASKθ-Myc #27 overexpressors were exposed to a light pulse of 8 h to stimulate germination and were subsequently wrapped in aluminium foil. After 4 days a picture of representative plants was taken.

**Figure S3.** Specificity of the anti-ASKθ antibody. ^35^S-methionine-labelled *in vitro*-translated proteins ASKθ, ASKβ and ASKη (BIN2) (lanes 4–6, respectively), and immunoprecipitations of the *in vitro*-translated proteins ASKθ, ASKβ and ASKη (BIN2) with an anti-ASKθ antibody (lanes 1–3, respectively).

**Figure S4.** Subcellular localization of ASKθ and BEH2. Fluorescence microscopy pictures (left) of guard and hypocotyl cells of 35S::ASKθ-YFP and 35S::BEH2-CFP transgenic *Arabidopsis thaliana* plants. Bright field pictures (middle) and overlays (right) are included for comparison. All pictures were taken at the same magnification. The bar represents 10 μm.

**Figure S5.** ASKθ-mediated *in vivo* phosphorylation of BES1 and BZR1. *Arabidopsis* protoplast cells were co-transformed with expression constructs for CFP-tagged BES1 (a) or BZR1 (b) and increasing amounts of Myc-tagged ASKθ. For BES1-CFP and BZR1-CFP, 5 μg of DNA was used; for ASKθ, 0.1 μg (lane 2), 0.3 μg (lane 3), 1 μg (lane 4) and 3 μg (lanes 5 and 6) were transformed. BES1-CFP and BZR1-CFP were detected by western blot analysis with an anti-GFP antibody. The positions of the phosphorylated and unphosphorylated forms of BES1 and BZR1 are indicated.

**Figure S6.** Interaction study of ASKθ and BIN2 with the family of BES1/BZR1-like transcription factors. ASKθ and BIN2 cloned into the binding domain vector pGBT9, and BES1, BZR1 and BEH1-4 cloned into the activation domain vector pACT2 were co-transformed into the *Saccharomyces cerevisiae* strain PJ69-4A. Empty vectors were used as controls. The β-galactosidase activity of protein extracts was measured using ONPG as a substrate and calculated as nmol o-nitrophenol liberated in 1 h mg^−1^ of protein. The means and standard deviations were calculated from 3 independent experiments.

**Table S1.** List of primers.

Please note: As a service to our authors and readers, this journal provides supporting information supplied by the authors. Such materials are peer-reviewed and may be re-organized for online delivery, but are not copy-edited or typeset. Technical support issues arising from supporting information (other than missing files) should be addressed to the authors.
