# Supplementary figures and images for: ASKθ, a group-III Arabidopsis GSK3, functions in the brassinosteroid signalling pathway
Source: Plant J. 2010 Feb 25;62(2):215–23. doi: 10.1111/j.1365-313X.2010.04145.x (PMC2881309; doi:10.1111/j.1365-313X.2010.04145.x)

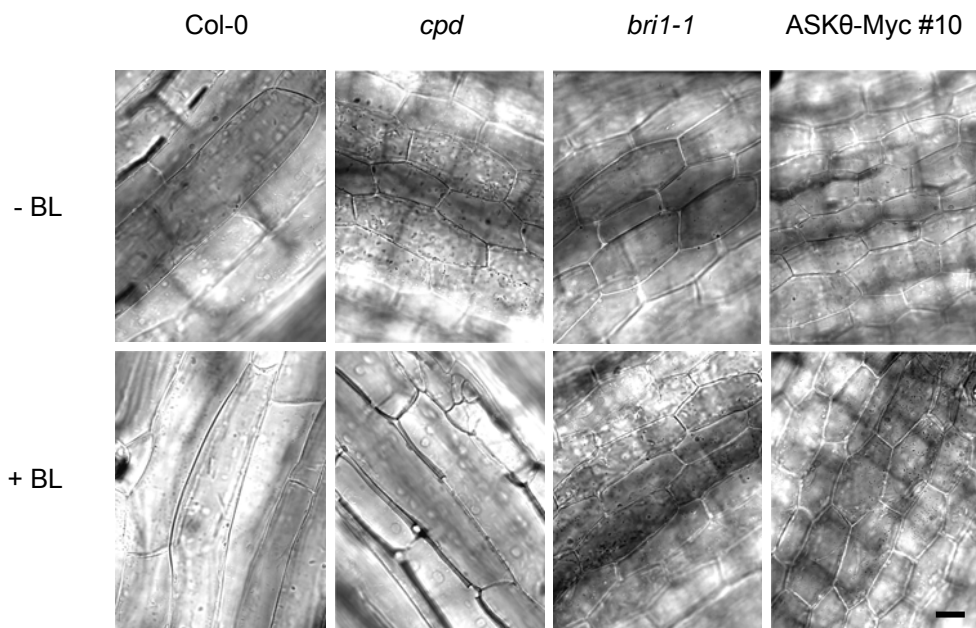

Figure S1

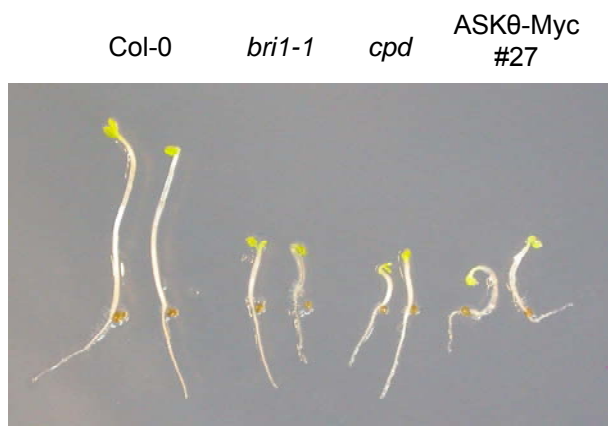

Figure S2

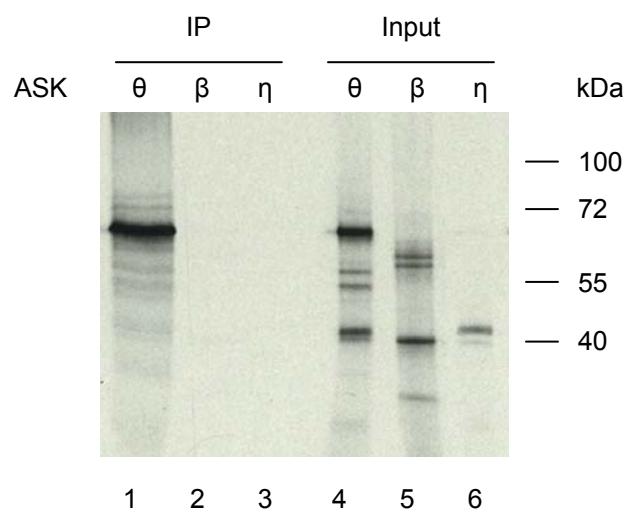

Figure S3

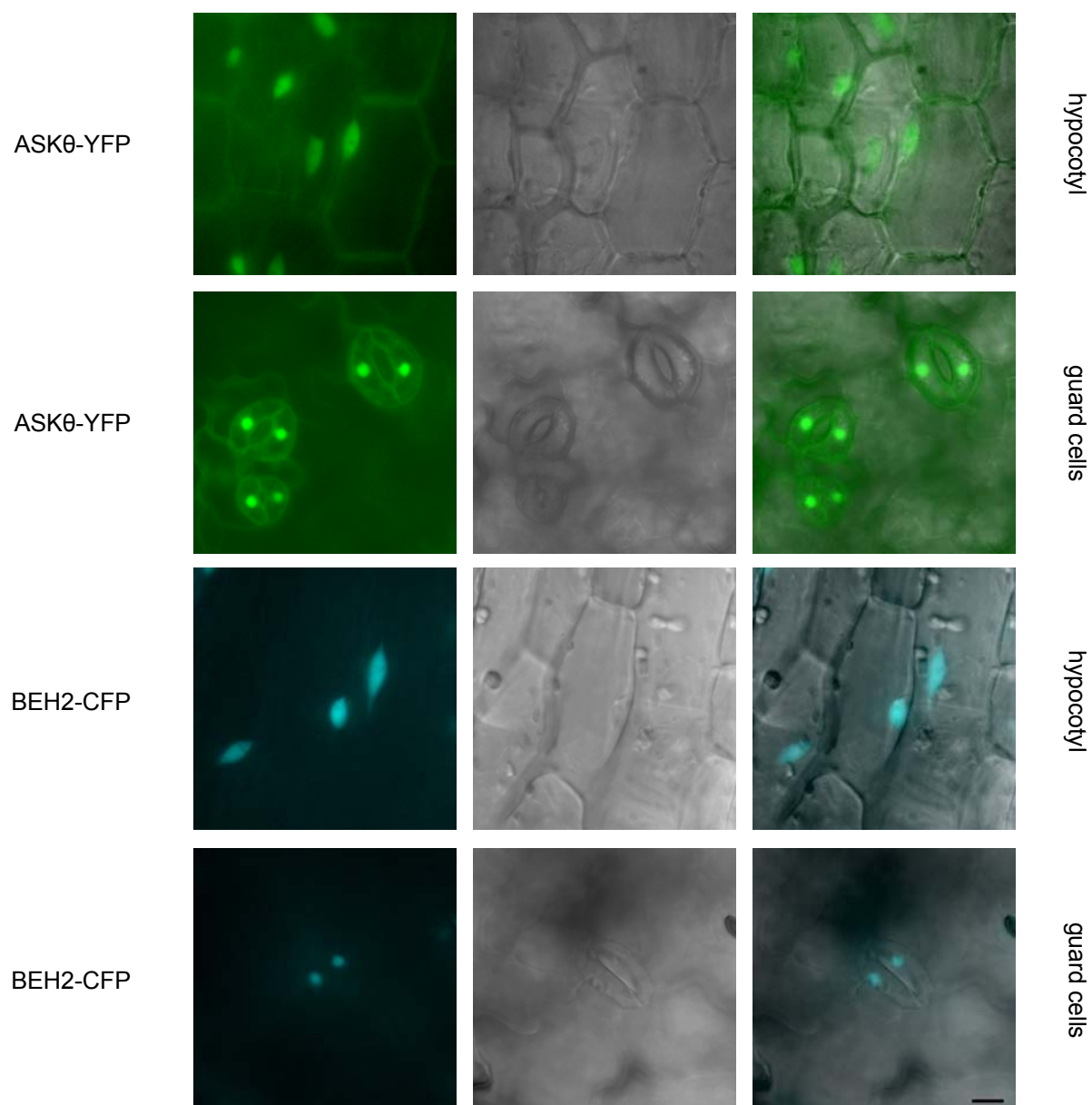

Figure S4

(a)

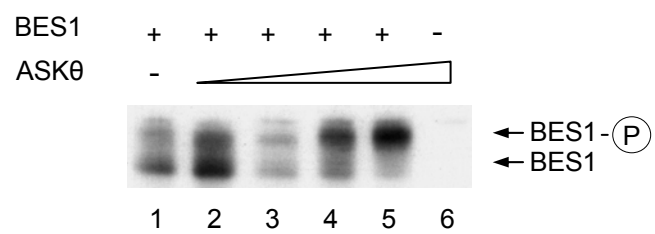

(b)

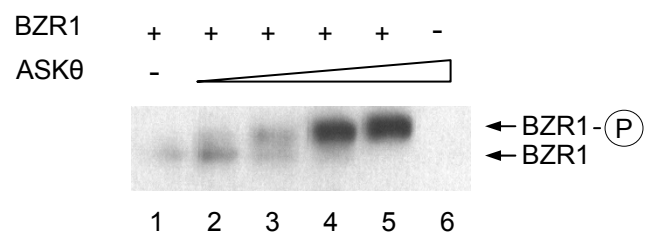

Figure S5

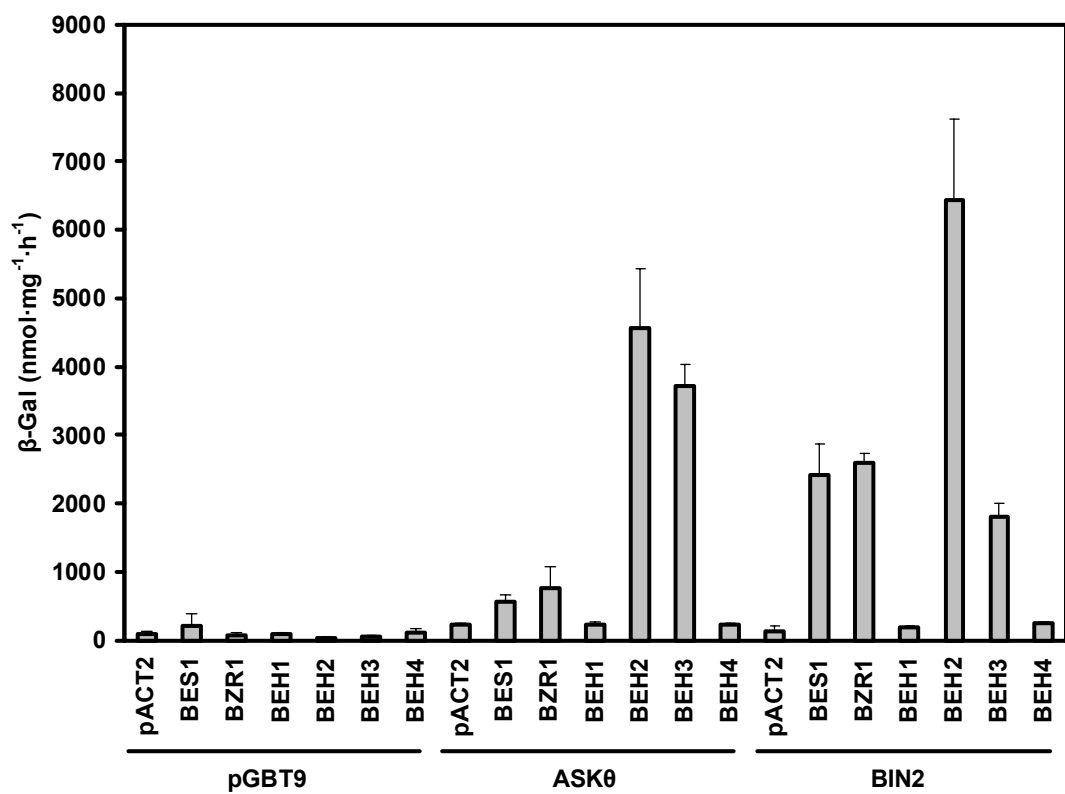

Figure S6

Supplement: Supplementary file 1 [file tpj0062-0215-SD1.pdf]
