# Supplementary material for: ASKθ, a group-III Arabidopsis GSK3, functions in the brassinosteroid signalling pathway
Source: Plant J. 2010 Feb 25;62(2):215–23. doi: 10.1111/j.1365-313X.2010.04145.x (PMC2881309; doi:10.1111/j.1365-313X.2010.04145.x)
Supplement: Supplementary file 2 [file tpj0062-0215-SD2.pdf]

Supplementary Table 1: Primers used in this study

| Primer | Sequence (5´ to 3´) | Used for |
|---|---|---|
| ASKθ fwd | TAGGATCCAGATGAACGTGATGCGTCGTCTCAAG | Cloning of the ASKθ coding sequence |
| ASKθ rev | GGATCCTAGCGGCCGCAAGAGCTACTTCCCGTTCCCTGGC | |
| ASKβ fwd | GGATCCTTATGAATGTGGTGCGGAGATTAAC | Cloning of the ASKβ coding sequence |
| ASKβ rev | GGATCCGCGGCCGCATTTCCTTGCATGCTCAGGTATTA | |
| BIN2 fwd | GGATCCTTATGGCTGATGATAAGGAGATGCC | Cloning of the BIN2 coding sequence |
| BIN2 rev | GGATCCGCGGCCGCTAGTTCCAGATTGATTCAAGAAGCT | |
| BES1 fwd | GATATCCATGACGTCTGACGGAGCA | Cloning of the BES1 coding sequence |
| BES1 rev | GCGGCCGCTACTATGAGCTTTACCATTTCC | |
| BZR1 fwd | GATATCCATGACTTCGGATGGAGCTACGTCGAC | Cloning of the BZR1 coding sequence |
| BZR1 rev | GCGGCCGCAACCACGAGCCTTCCCATTTCCAAGTGT | |
| BEH2 fwd | GATATCCATGGCCGCCGGAGGAGGAGGA | Cloning of the BEH2 coding sequence |
| BEH2 rev | GCGGCCGCAGCATCTGGCTTTAGTGCCA | |
| $P_{ASK\theta}$ fwd | CTCGAGGGGCCTACAACAATAT | Cloning of an ASKθ 2007 bp promoter fragment |
| $P_{ASK\theta}$ rev | CCATGGTCGCTTTATTCACCAA | |
| $P_{BIN2}$ fwd | CTCGAGACGGTTGGCTGTTAGTT | Cloning of the BIN2 1992 bp promoter fragment |
| $P_{BIN2}$ rev | CCATGGGCATAGAGACACAGAGA | |
| ACT3 fwd | ATGGTTAAGGCTGGTTTTGC | Semiquantitative RT-PCR |
| ACT3 rev | AGCACAATACCGGTAGTACG | |
| CBP fwd | GCATCTACAACGGTTTACAT | Semiquantitative RT-PCR |
| CBP rev | GTCGTTTCCGATGGTAGTCT | |
| DWF4 fwd | GTCATCCTCAGGAAGTGGTAGT | Semiquantitative RT-PCR |
| DWF4 rev | TACAGAATACGAGAAACCCTAATAG | |
| Br6Ox2 fwd | CCAAGAGTTTCTGCACCAAAAGGATAC | Semiquantitative RT-PCR |
| Br6Ox2 rev | AGGTTAATTAGCCCTACAAAATGACCC | |
| BEE3 fwd | TCGACGAGGGAAAATAAAC | Semiquantitative RT-PCR |
| BEE3 rev | TCAAAGGGTCCACGATG | |
